# Supplementary material for: DELE1 promotes translation-associated homeostasis, growth, and survival in mitochondrial myopathy
Source: bioRxiv. 2024 Feb 29:2024.02.29.582673. Preprint. [Version 1] doi: 10.1101/2024.02.29.582673 (PMC10962736; doi:10.1101/2024.02.29.582673)

bioRxiv preprint doi: https://doi.org/10.1101/2024.02.29.582673; this version posted February 29, 2024. The copyright holder for this preprint (which was not certified by peer review) is the author/funder. This article is a US Government work. It is not subject to copyright under 17 USC 105 and is also made available for use under a CC0 license.

Supplemental Figure Legends

Supplemental Figure 1. OMA1-DELE1 pathway mediates the integrated stress response in brown adipose tissue under cold stress.

(A) H&E-stained section of brown adipose tissue from *Dele1* KO, *Oma1* KO, and WT littermates (of *Dele1* KO) subjected to cold stress in (Fig. 1H) shows reduction in brown adipose lipid droplets in all genotypes, which appear as unstained spheres, after cold stress.

(B) Immunoblot showing OPA1 cleavage by OMA1 in WT and *Dele1* KO but not *Oma1* KO mice that were analyzed in (Fig. 1I).

(C) Scheme depicts the logic used for defining DELE1-dependent DEGs from global gene expression data.

(D) Volcano plot of microarray data from experiment in (Fig. 1I-J) comparing gene expression changes between *Dele1* KO and WT littermates left at room temperature (RT). No significant gene changes were observed. N=7 mice per group.

Supplemental Figure 2. DELE1 mt-ISR promotes survival in diverse models of mitochondrial stress.

(A) Grip strength (left) and a composite phenotype score (right) of C10 G58R; *Dele1* KO mice and littermates at P28. Composite phenotype score is comprised of ledge test, hindlimb clasping, gait test, and kyphosis.

(B) Survival analysis of C10 G58R mice with and without nutritional support involving hand feeding twice per day.

(C) Simple linear regression between gastrocnemius muscle fiber cross sectional area (CSA) and body weight for of C10 G58R; *Dele1* KO mice and littermates.

bioRxiv preprint doi: https://doi.org/10.1101/2024.02.29.582673; this version posted February 29, 2024. The copyright holder for this preprint (which was not certified by peer review) is the author/funder. This article is a US Government work. It is not subject to copyright under 17 USC 105 and is also made available for use under a CC0 license.

(D) Heart to body weight ratio for C10 S59L mice; *Dele1* KO mice and littermates at P140.

(E) Heart to body weight ratio for *Tfam* mKO mice; Dele1 KO mice and littermates at P56.

(F) Correlation between age at stress onset and DELE1 survival benefit among the models. * indicates genotypes for which some lifespan estimates were determined from prior studies (Nguyen *et al*, 2022; Shammas *et al*, 2022).

(G) Primary fibroblasts from *Opa1*$^{\Delta s1/\Delta s1}$ mice treated with CCCP 20 μM or vehicle only for 16 hrs. The *c* and *e* bands generated by OMA1 cleavage (from *a* and *b*, respectively) are reduced at baseline and following uncoupling with CCCP; the *b* band is also relatively retained with CCCP, together demonstrating that L-OPA1$^{\Delta s1/\Delta s1}$ (*a* and *b* bands) is resistant to OMA1 cleavage.

(H) Composite phenotype score of C10 G58R; *Opa1*$^{\Delta s1/\Delta s1}$ mice and littermates at 13 weeks.

(I) Immunoblot compares OPA1 cleavage by OMA1 and elevation of the mt-ISR marker protein MTHFD2 in C10 G58R animals with or without OMA1 or DELE1 that survived to 14 – 16 weeks. Lysates from *Oma1* KO animals are from samples that were previously generated and appeared in (Shammas *et al*, 2022).

Supplemental Figure 3. Comparison of OXPHOS subunit expression in heart mitochondria from diverse models of mitochondrial myopathy/cardiomyopathy.

(A) Scatterplots depicting relative abundance of OXPHOS complexes I – V subunits, mito-ribosome, and Coenzyme Q from the indicated genotypes. Data are from the same datasets represented in (Fig. 3F – H), replotted to compare disease models. For statistics, a one-way ANOVA was performed followed by post-hoc testing, corrected for the multiple comparisons depicted within the graph with Dunnett's test. All values are relative to littermate controls except for C2/C10 DKO which are matched to unrelated age-matched controls. Data from these proteomics datasets also appear in Fig. 5G - I.

bioRxiv preprint doi: https://doi.org/10.1101/2024.02.29.582673; this version posted February 29, 2024. The copyright holder for this preprint (which was not certified by peer review) is the author/funder. This article is a US Government work. It is not subject to copyright under 17 USC 105 and is also made available for use under a CC0 license.

(B) Enrichment analysis mitochondrial proteins that significantly changed in *Tfam* mKO vs. control mitochondria isolated from hearts, using Tier 3 MitoPaths from MitoCarta 3.0.

Supplemental Figure 4. TEM of myocardium and ultrastructural features of mitochondria in C10 G58R on Dele1+/- and Dele1 KO backgrounds.

(A) Kernel density plots showing distribution of mitochondrial areas for indicated genotypes, measured from TEM images of heart mitochondria. Median values and, in parentheses, interquartile ranges are reported adjacent to curves. N = 2 animals per genotype except for *Tfam* mKO; *Dele1* KO, where only 1 animal was available. > 600 mitochondria were measured per animal.

(B) Bar graph comparing the areas of segmented and non-segmented types of electrolucent mitochondria that were obtained from analysis of C10 G58R animals and littermates in (A). Statistics were performed using Mann-Whitney test.

(C - E) Representative TEM images acquired at 2000x direct magnification show areas of myocardium of indicated genotype used for analysis of mitochondria. Scale bar = 5 µm.

(F) Image of the subarea boxed yellow in D, acquired at 5000x direct magnification and representative of the images used to quantify ultrastructural features of mitochondria detailed in Figure 4. Scale bar = 2.5 µm.

(G) Examples of inclusions observed in C10 G58R mutant mitochondria (black arrows).

(H) Examples of two types of electrolucent mitochondria characterized by an enlarged matrix area absent of electron-dense substance and fewer cristae. (Top) A uniformly electrolucent mitochondrion. (Bottom) a segmented mitochondrion that has an electrolucent part (white arrow) separated from a

bioRxiv preprint doi: https://doi.org/10.1101/2024.02.29.582673; this version posted February 29, 2024. The copyright holder for this preprint (which was not certified by peer review) is the author/funder. This article is a US Government work. It is not subject to copyright under 17 USC 105 and is also made available for use under a CC0 license.

portion of normal-looking matrix and cristae by a cut-through cristae. Open black arrowhead indicates the junction between electrolucent and normal portions of the segmented mitochondria.

(I) Mitochondria that are fully wrapped by electron-dense phagosome membranes (black arrows).

(J) Mitochondria with ruptured OMMs. Open white arrowheads indicate sites where the intact IMM is visible, but OMM is absent. Scale bar in J = 500 nm and applies to G-J.

Supplemental Figure 5. TEM of myocardium and ultrastructural features of mitochondria in C2/C10 DKO.

(A-B) Representative TEM images acquired at 2000x direct magnification show areas of myocardium of indicated genotype used for analysis of mitochondria. Scale bar = 5 µm.

(C) Image of the subarea boxed yellow in B, acquired at 5000x direct magnification and representative of the images used to quantify ultrastructural features of mitochondria detailed in Figure 4. Scale bar = 2.5 µm.

(D) Examples of inclusions observed in C2/C10 DKO mitochondria (black arrows). Scale bar = 500 nm.

(E) Examples of electrolucent mitochondria characterized by an enlarged matrix area absent of electron-dense substance and fewer cristae. Scale bar = 500 nm.

Supplemental Figure 6. TEM of myocardium and ultrastructural features of mitochondria in C10 S59L on Dele1+/- and Dele1 KO backgrounds.

(A-C) Representative TEM images acquired at 2000x direct magnification show areas of myocardium of indicated genotype used for analysis of mitochondria. Scale bar = 5 µm.

bioRxiv preprint doi: https://doi.org/10.1101/2024.02.29.582673; this version posted February 29, 2024. The copyright holder for this preprint (which was not certified by peer review) is the author/funder. This article is a US Government work. It is not subject to copyright under 17 USC 105 and is also made available for use under a CC0 license.

(D) Image of the subarea boxed yellow in B, acquired at 5000x direct magnification and representative of images used to quantify ultrastructural features of mitochondria detailed in Figure 4. Scale bar = 2.5 µm.

(E) Examples of mitochondria that are partially or fully enclosed by electron-dense phagosome membranes (black arrows). Open black arrowhead indicates a portion of the mitochondria that is not enclosed. Partially enclosed mitochondria with ruptured OMMs were also observed. Open white arrowheads indicate sites where the intact IMM is visible, but the OMM is absent.

(F) Examples of mitochondria with ruptured OMMs. Open white arrowheads indicate sites where the intact IMM is visible, but an OMM is absent. Scale bar in F = 500 nm and applies to E.

(G) Serial sections through a 250 nm diameter mitochondrion show that it is a spherical nano-mitochondrion spanning fewer than five 60-nm sections (< 300 nm in Z). Top row shows the five serial sections without colorization, bottom row shows the same serial sections with the nano-mitochondrion shaded yellow. Yellow dotted lines indicate absence of the mitochondrion in neighboring serial sections. Scale bar = 200 nm.

(H) Five serial sections of 60-nm thickness show a 100 nm-wide tubule-shaped mitochondrion. Top row shows five serial sections through the tubular nano-mitochondrion, bottom row shows the same serial sections with the tubular nano-mitochondrion shaded yellow. The yellow dotted lines indicate absence of the mitochondrion in the neighboring section. Scale bar = 200 nm.

Supplemental Figure 7. TEM of myocardium and ultrastructural features of mitochondria in *Tfam* mKO on *Dele1*+/- and *Dele1* KO backgrounds.

bioRxiv preprint doi: https://doi.org/10.1101/2024.02.29.582673; this version posted February 29, 2024. The copyright holder for this preprint (which was not certified by peer review) is the author/funder. This article is a US Government work. It is not subject to copyright under 17 USC 105 and is also made available for use under a CC0 license.

(A-C) Representative TEM images acquired at 2000x direct magnification show areas of myocardium of indicated genotype used for analysis of mitochondria. Yellow star in B indicates a myocyte with milder structural phenotype compared to neighboring myocytes, illustrating the observed mosaicism of the phenotype. Scale bar = 5 µm.

(D) Image of the subarea boxed yellow in B, acquired at 5000x direct magnification and representative of images used to quantify ultrastructural features of mitochondria detailed in Figure 4. Scale bar = 2.5 µm.

(E) *Tfam* mKO mitochondria displayed populations of closely aligned “stacked” cristae (black arrows) and sparse areas filled with a granular matrix material and few cristae (open white arrowheads). Scale bar = 500 nm.

(F) Examples of crumpled cristae (white arrows) that occurred in *Tfam* mKO mitochondria. Scale bar = 500 nm.

(G) Stacked cristae boxed in E and crumpled cristae boxed in F are shown enlarged in G. Scale bar = 250 nm.

Supplemental Figure 8. DELE1 mt-ISR transcriptional response in heart is similar in response to diverse mitochondrial stressors.

(A) Heat map of Log_2FC for DELE1-dependent DEGs detected in hearts of two out of three myopathy/cardiomyopathy models.

(B) Venn diagram showing intersection of stress-induced DEGs in heart among the 3 models of myopathy/cardiomyopathy.

bioRxiv preprint doi: https://doi.org/10.1101/2024.02.29.582673; this version posted February 29, 2024. The copyright holder for this preprint (which was not certified by peer review) is the author/funder. This article is a US Government work. It is not subject to copyright under 17 USC 105 and is also made available for use under a CC0 license.

(C) Plot depicts the number of stress induced DEGs ≥ indicated cutoffs for percent DELE1 dependence.

(D) Venn diagram showing intersection of DELE1-dependent DEGs and mitochondrial stress-induced DEGs that are ≥ 50% DELE1-dependent.

Supplemental Figure 9. DELE1 mt-ISR transcriptional response in heart has variable effects on *Gpx4* expression and mediates most transcriptionally driven increases in mitochondrial proteins involved in proteostasis.

(A-B) GPX4 protein levels from heart whole cell lysate (WCL) of indicated genotypes.

(C) *Gpx4* mRNA levels measured in microarray experiments, described in (Fig 5C).

(D) GPX4 mitochondrial protein levels measured in proteomics experiments, described in (Fig 5G and H).

(E - F) Scatterplot compares RNA log_2FC for *Tfam* mKO vs. control (in the presence of DELE1) and mitochondrial protein log_2FC for *Tfam* mKO vs. control animals in the presence of DELE1 (left) or the absence of DELE1 (right).

(G – H) Bar graphs showing protein and RNA fold changes for proteins annotated as proteases and chaperones in MitoCarta3.0, from experiments described in (Fig. 5C, G, and H).

Supplemental Figure 10. Metabolomics from hearts of C10 G58R and *Tfam* mKO models of mitochondrial myopathy/cardiomyopathy.

(A - C) Volcano plots of metabolites identified in targeted (B and C) or untargeted (A) metabolomics experiments and enrichment analysis among KEGG metabolite sites for the DELE1-dependent

bioRxiv preprint doi: https://doi.org/10.1101/2024.02.29.582673; this version posted February 29, 2024. The copyright holder for this preprint (which was not certified by peer review) is the author/funder. This article is a US Government work. It is not subject to copyright under 17 USC 105 and is also made available for use under a CC0 license.

metabolites. Only named features in the untargeted metabolomics data are plotted in (A). DELE1-dependent metabolites are in blue, with amino acids bolded.

Supplemental Figure 11. Transcriptomics from liver from C10 G58R mice with either *Dele1* KO (P28) or *Oma1* knockdown using an ASO (~1 year).

(A) Heat map depicts the OMA1-dependent DEGs detected in liver from ~1 year old C10 G58R mice compared to P28 C10 G58R mice. No DELE1-dependent DEGs were detected at P28.

(B) Top gene ontology terms for the OMA1-dependent DEGs from livers of ~1 year old C10 G58R mice in (A).

Supplemental Figure 12. Transcriptomics from gastrocnemius skeletal muscle from P28 C10 G58R; *Dele1* KO mice and littermates.

(A) Heatmap of gene expression changes from gastrocnemius skeletal muscle for intersection of DELE1-dependent DEGs in gastrocnemius skeletal muscle and heart DELE1 mt-ISR signature. Bargraph on right represents percent DELE1 dependence.

(B) Heatmap of gene expression changes from gastrocnemius skeletal muscle for DELE1-dependent DEGs in gastrocnemius skeletal muscle that are not part of heart DELE1 mt-ISR signature. Bargraph on right represents percent DELE1 dependence.

(C) Heatmap of gene expression changes from gastrocnemius skeletal muscle for genes in the heart DELE1 mt-ISR signature that were not significantly DELE1-dependent in the gastrocnemius muscle. Bargraph on right represents percent DELE1 dependence.

bioRxiv preprint doi: https://doi.org/10.1101/2024.02.29.582673; this version posted February 29, 2024. The copyright holder for this preprint (which was not certified by peer review) is the author/funder. This article is a US Government work. It is not subject to copyright under 17 USC 105 and is also made available for use under a CC0 license.

(D) Bar graphs depicting mRNA changes for *Foxo1* and two canonical atrogenes, *Trim63* and *Fbox32*, measured in microarray data of gastrocnemius skeletal muscle lysates.

(E) Immunoblot of heart (top) and gastrocnemius muscle (bottom) from P28 C10 G58R; *Dele1* KO mice and their littermates, showing DELE1-dependent phosphorylation of eIF2α.

Supplemental Figure 13. The Dele1 mt-ISR prevents disruptions in translation-associated proteostasis.

(A) Representative immunofluorescence images of gastrocnemius muscle from C10 G58R; *Dele1* KO mice and littermates, showing fibers with confluent aggregates of ubiquitinated proteins co-localized with the aggregate-forming adaptor protein p62, suggesting proteostatic collapse (arrow heads) and individual aggregates staining positive for ubiquitinated protein and the aggregate-forming adaptor protein p62 (arrows) in high power (60X) images (bottom panels). Scale bars = 10 μm. Note: animals were not injected with puromycin in this experiment.

(B) Quantification of (B). Aggregates positive for both p62 and ubiquitinated protein immunofluorescence were counted in 10 high power (60X) fields. N = 3 mice per genotype with 29 or 30 fields counted total per sample. High-power (60X) field size is 132.58 μm X 132.58 μm.

(C) Representative immunofluorescence images of gastrocnemius muscle from P28 C10 G58R; Dele1 KO mice and littermates injected with puromycin 30 min prior to sacrifice as in (Fig. 7E). Muscle cross-sections were immunostained for ubiquitinated proteins (using the FK2 antibody) (green), puromycin (red), and LAMININ (blue). Arrow heads indicate muscle fibers containing many or confluent aggregates of ubiquitinated protein that were also co-stained for elevated puromycylated polypeptides. N = 1 mouse for each genotype except for P28 C10 G58R; Dele1 KO mice for which N = 3 mice. Scale bars = 20 μm.

bioRxiv preprint doi: https://doi.org/10.1101/2024.02.29.582673; this version posted February 29, 2024. The copyright holder for this preprint (which was not certified by peer review) is the author/funder. This article is a US Government work. It is not subject to copyright under 17 USC 105 and is also made available for use under a CC0 license.

(D) Quantification of myofiber cross-sectional area (CSA) in (Fig. 7E). The average CSA for Ub+ and Ub- myofiber is shown in graph separately for three mice (m1 – 3) in graph. N = 3 mice with 10 high power fields counted per mouse.

Supplemental Tables

Table 1. Transcriptomics from hearts of three myopathy/cardiomyopathy mouse model.

Table 2. Mitochondrial proteomics from hearts of three myopathy/cardiomyopathy mouse model.

Table 3. Metabolomics from hearts of two myopathy/cardiomyopathy mouse model, *Tfam* mKO and C10 G58R.

Supplemental Figure 1

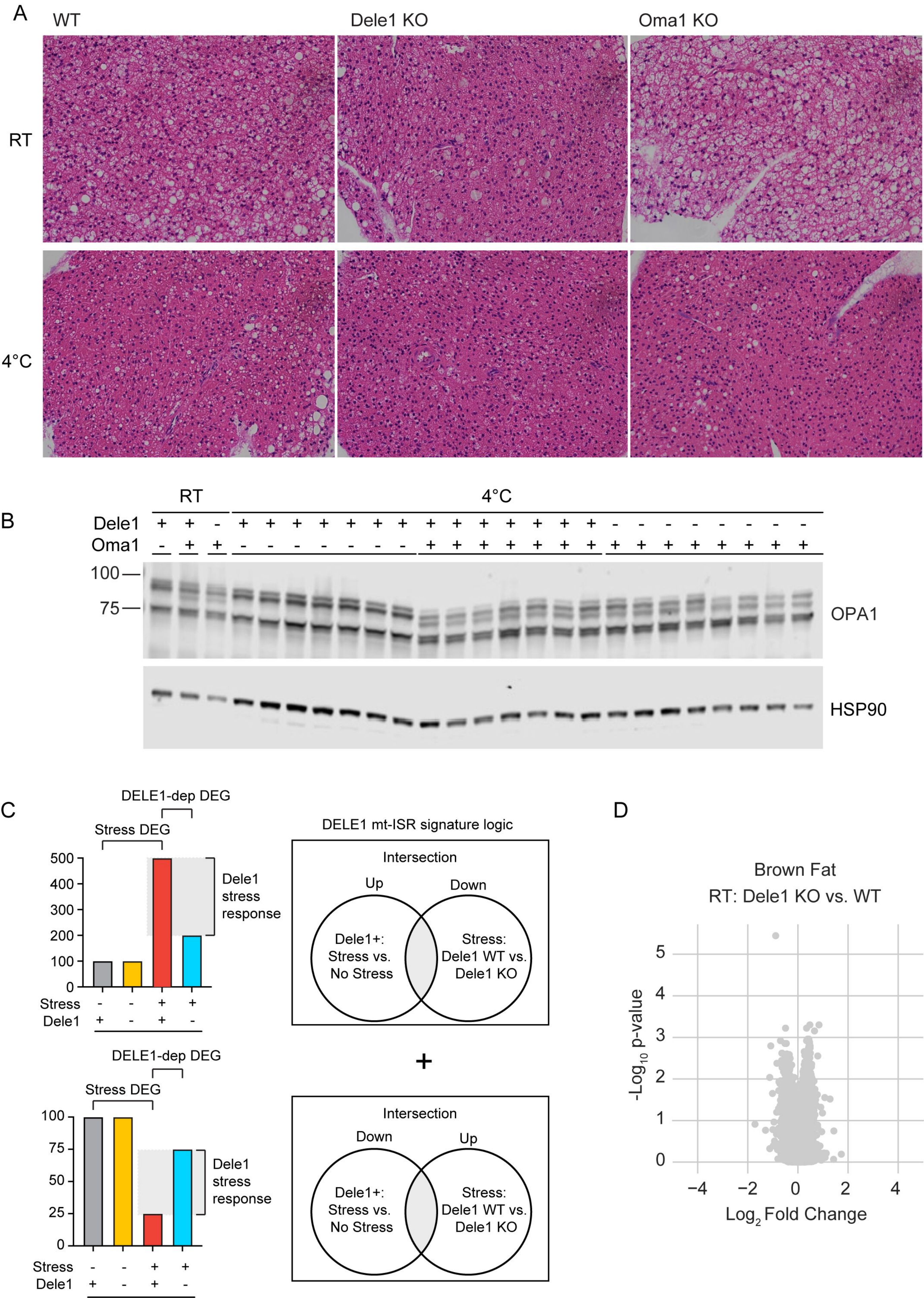

Supplemental Figure 2

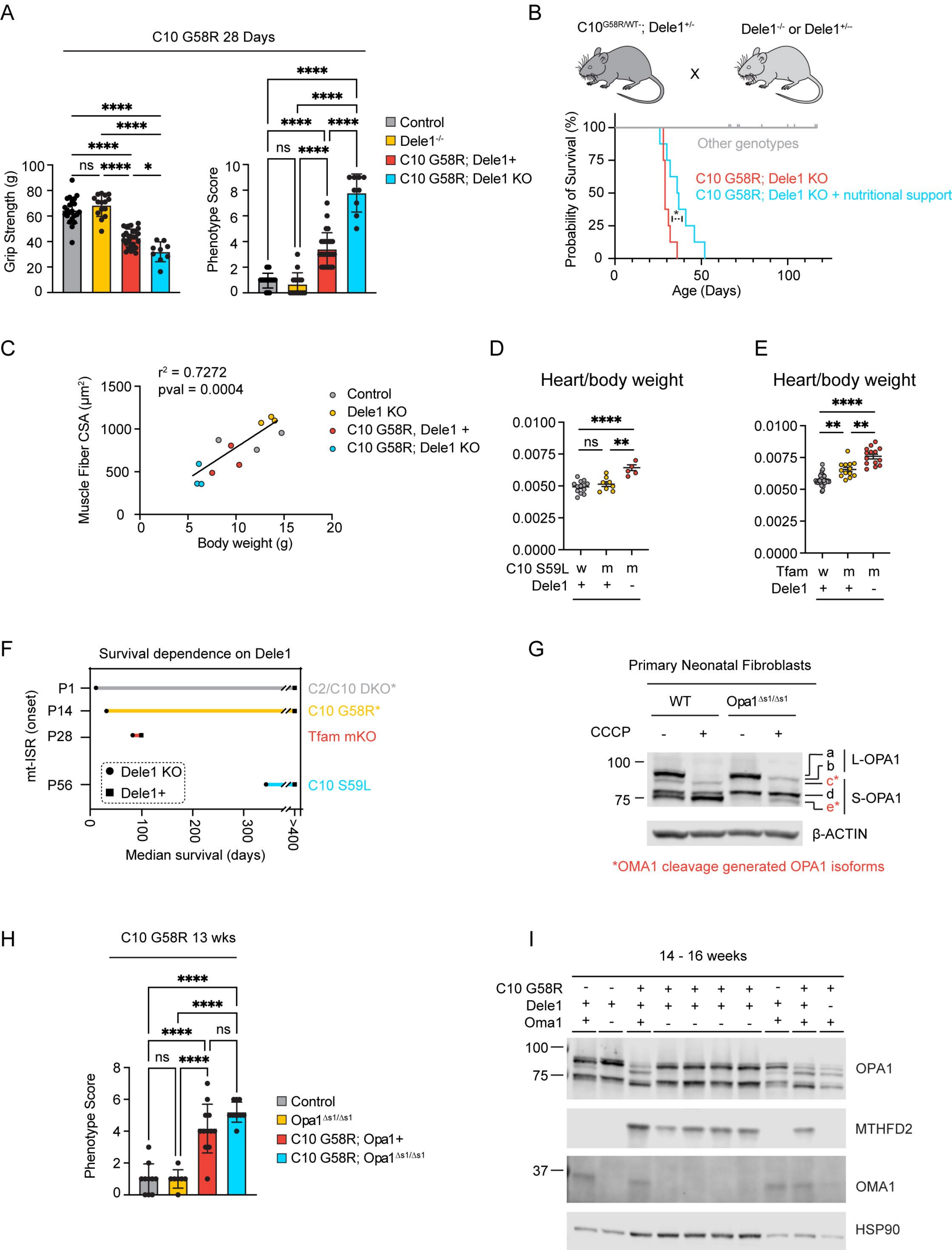

Supplemental Figure 3

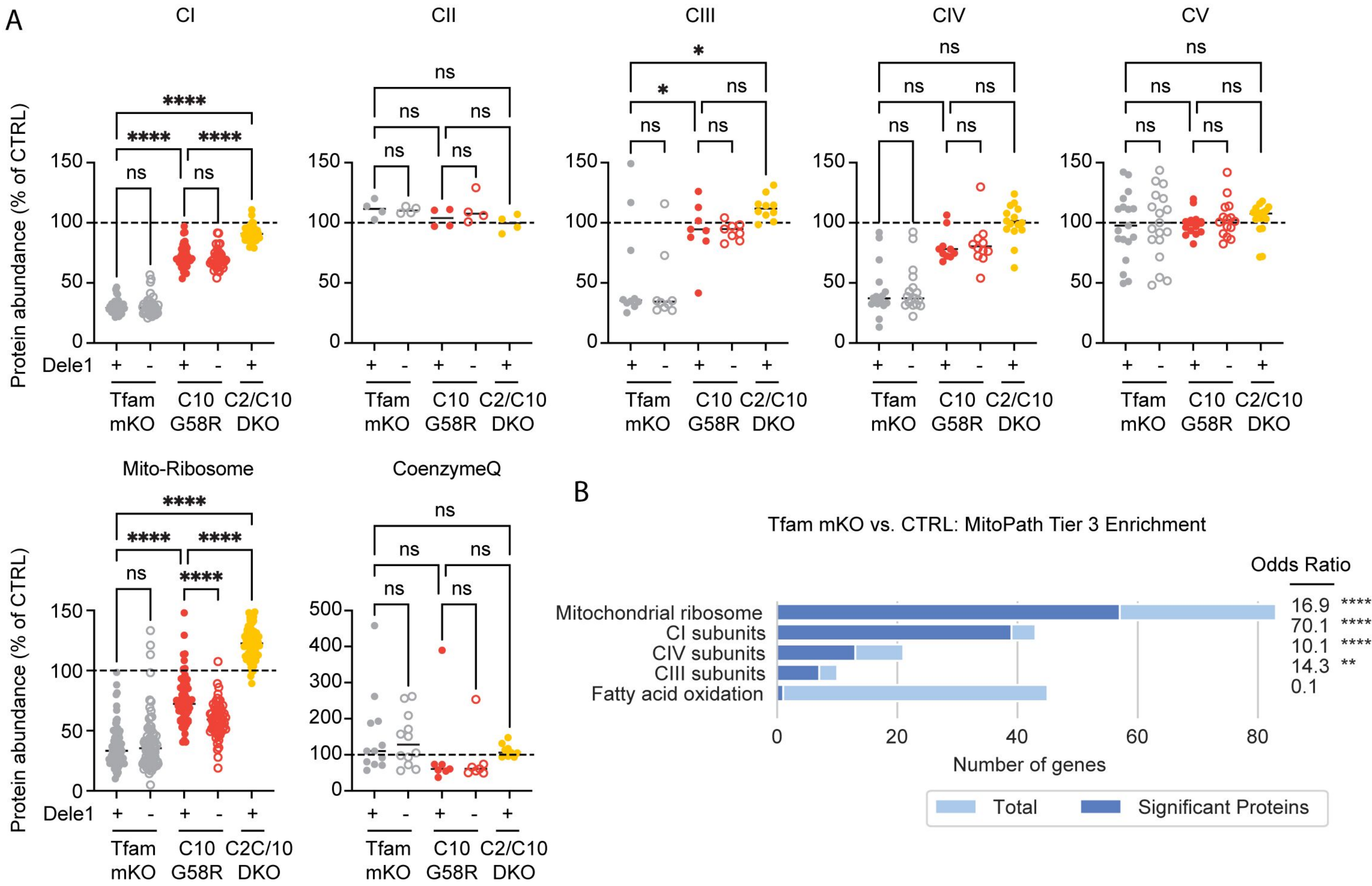

Supplemental Figure 4

bioRxiv preprint doi: https://doi.org/10.1101/2024.02.29.582673; this version posted February 29, 2024. The copyright holder for this preprint (which was not certified by peer review) is the author/funder. This article is a US Government work. It is not subject to copyright under 17 USC 105 and is also made available for use under a CC0 license.

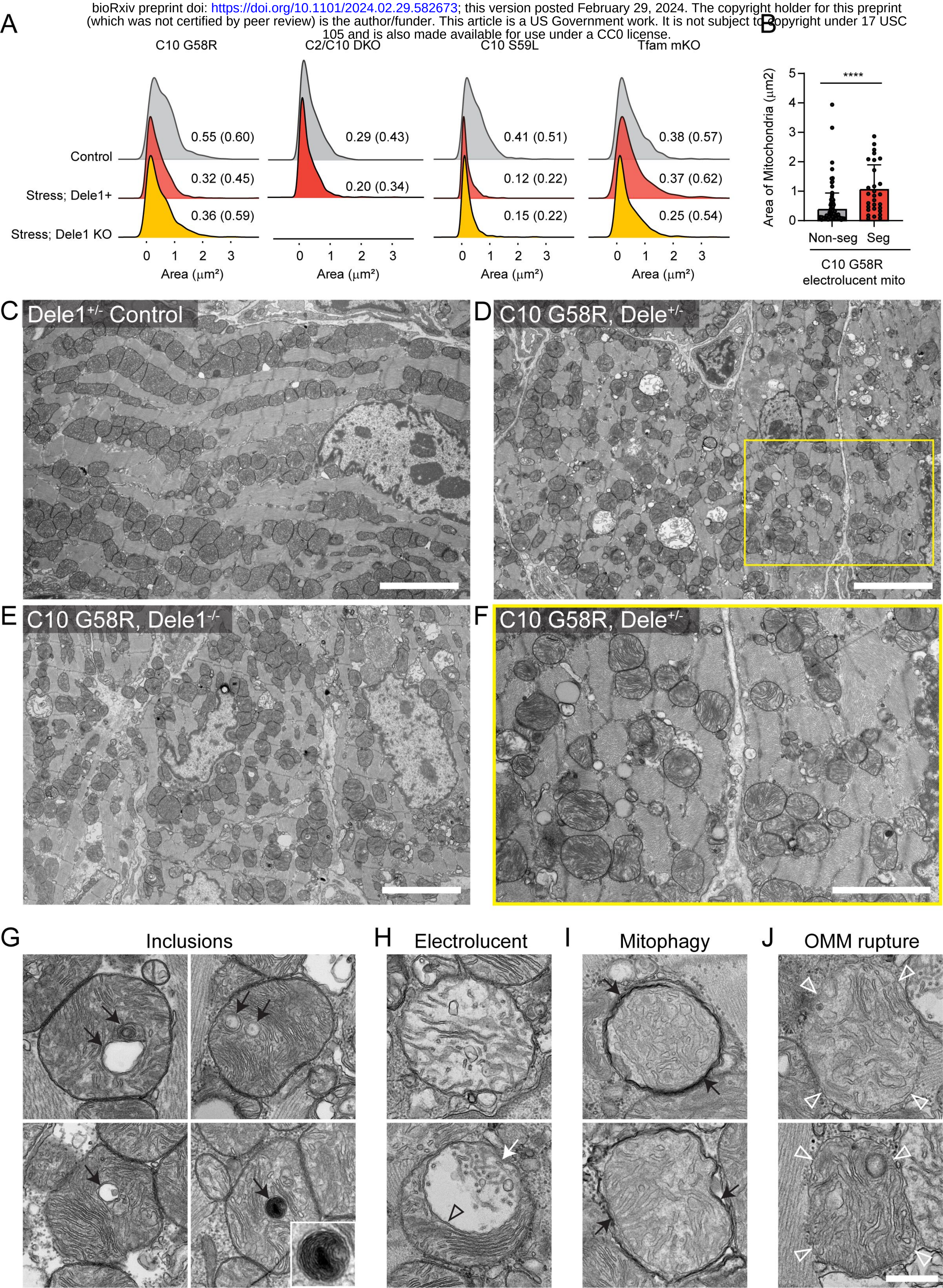

Supplemental Figure 5

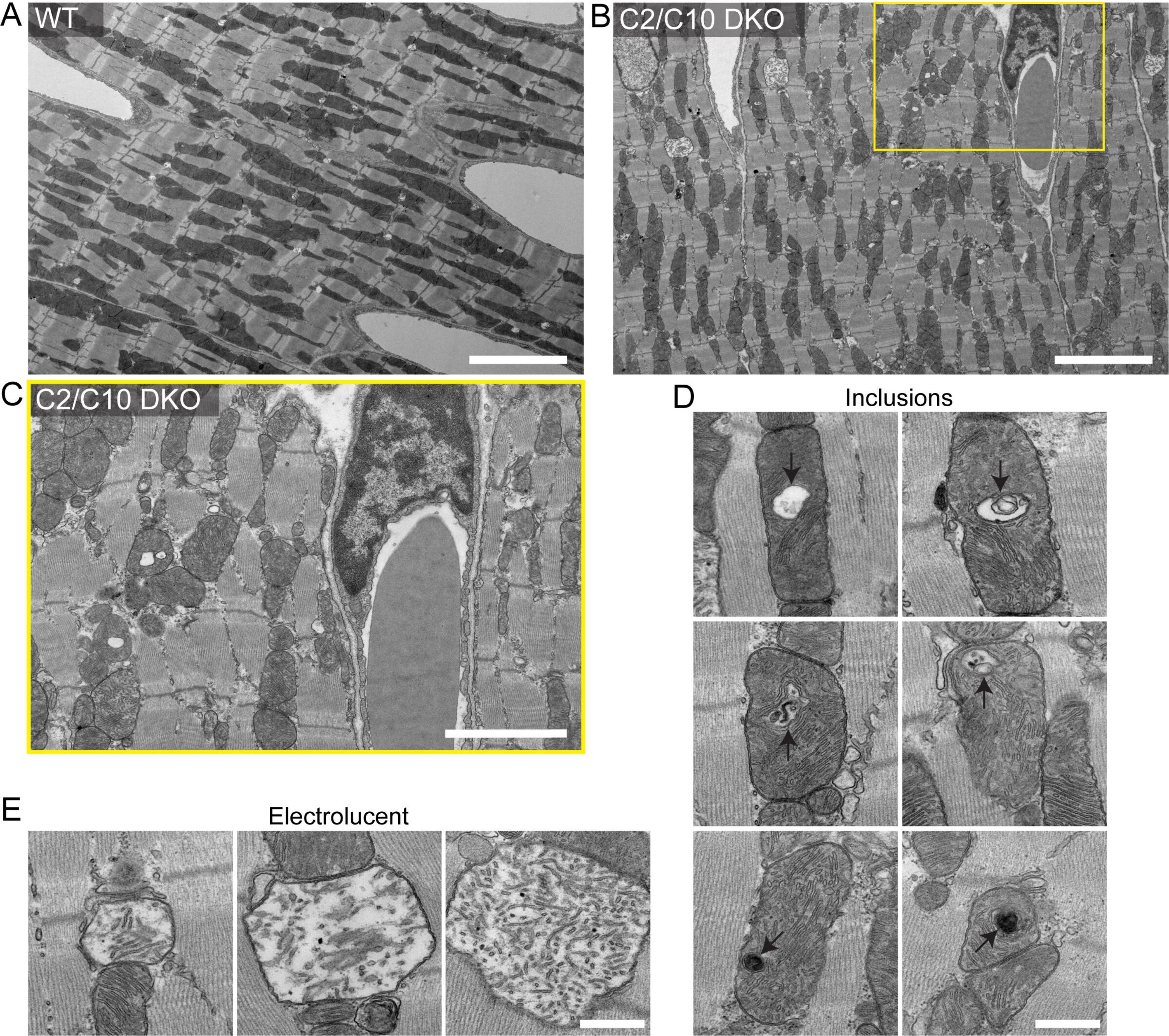

Supplemental Figure 6

bioRxiv preprint doi: https://doi.org/10.1101/2024.02.29.582673; this version posted February 29, 2024. The copyright holder for this preprint (which was not certified by peer review) is the author/funder. This article is a US Government work. It is not subject to copyright under 17 USC 105 and is also made available for use under a CC0 license.

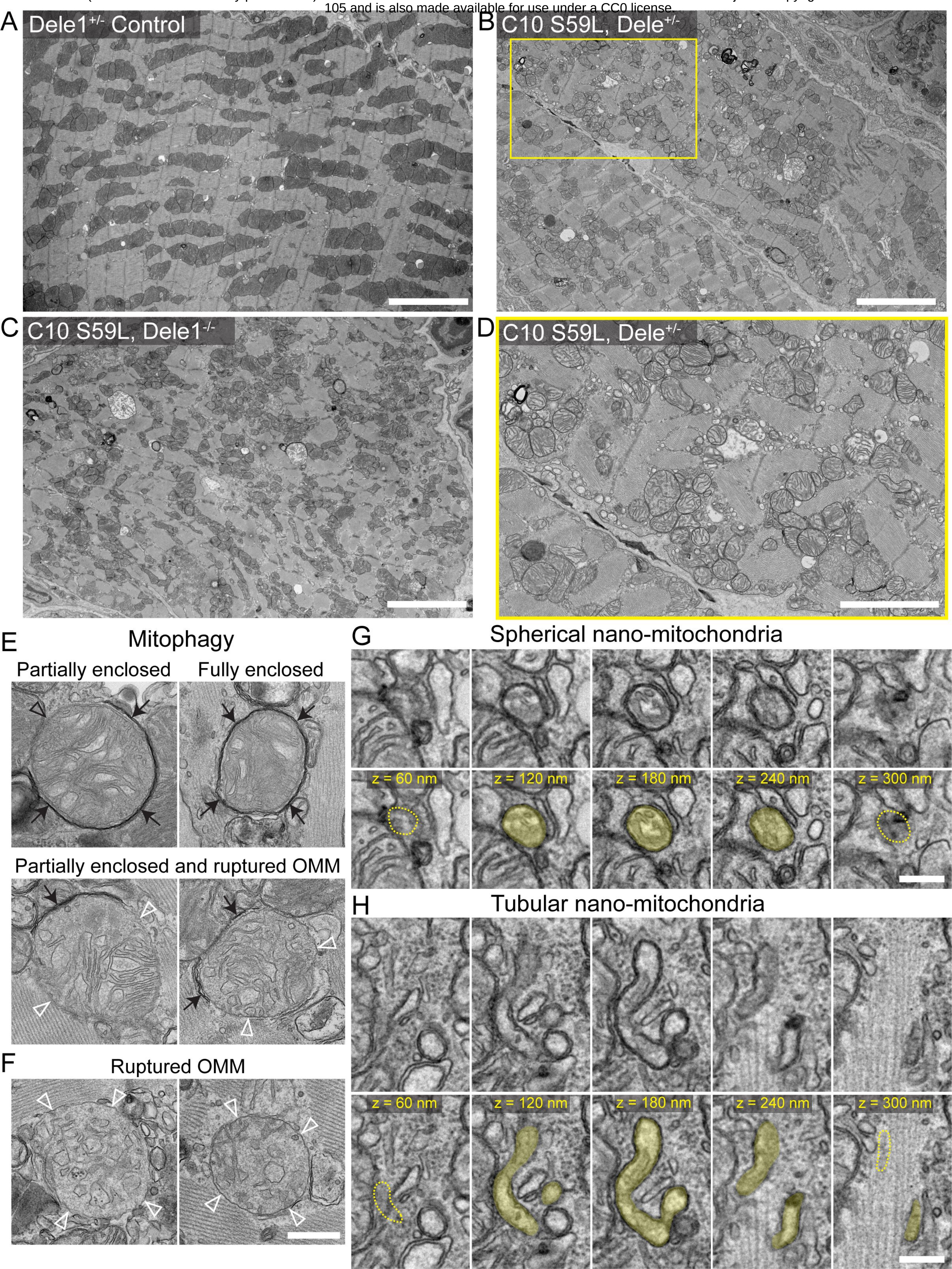

Supplemental Figure 7

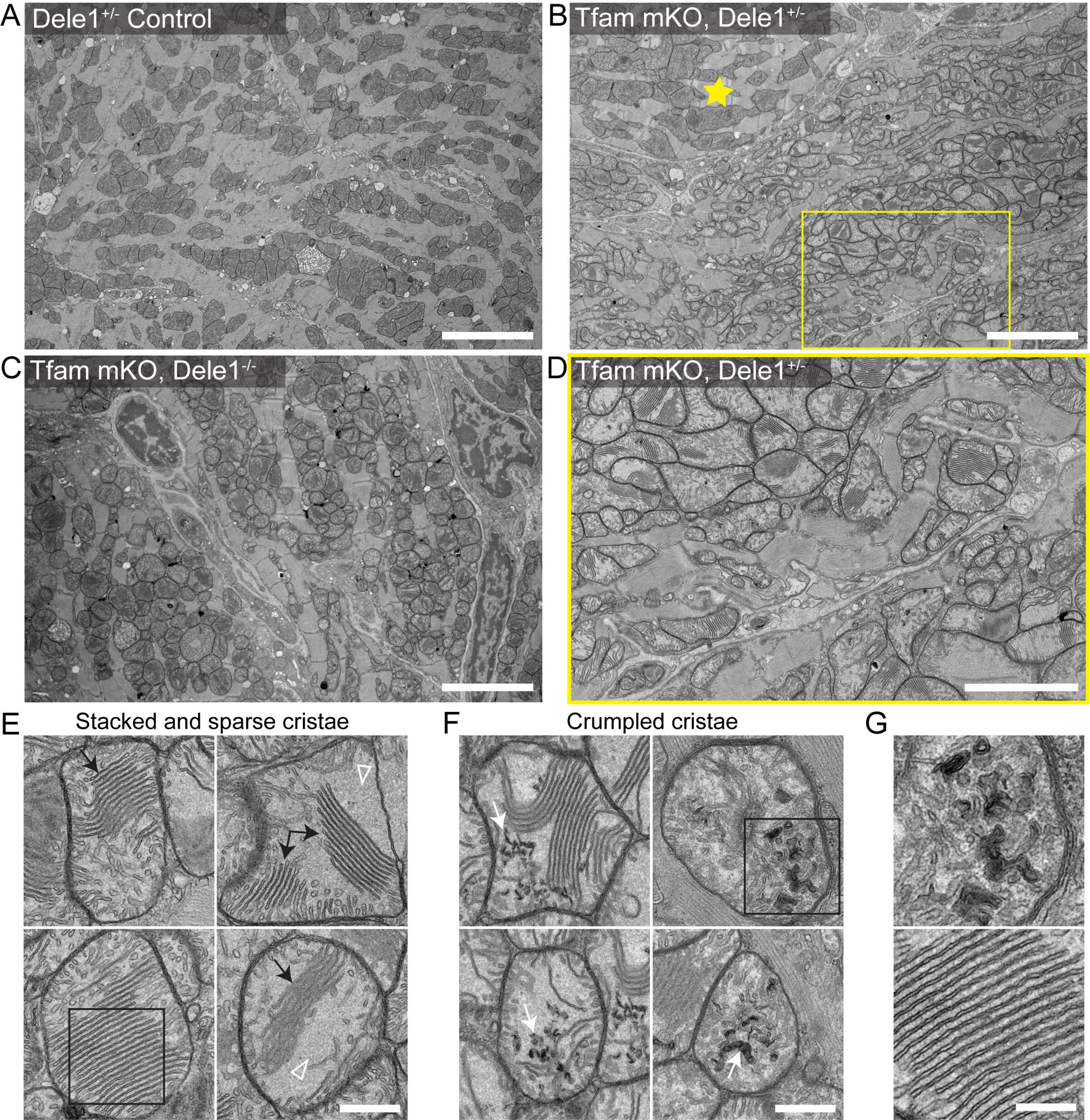

Supplemental Figure 8

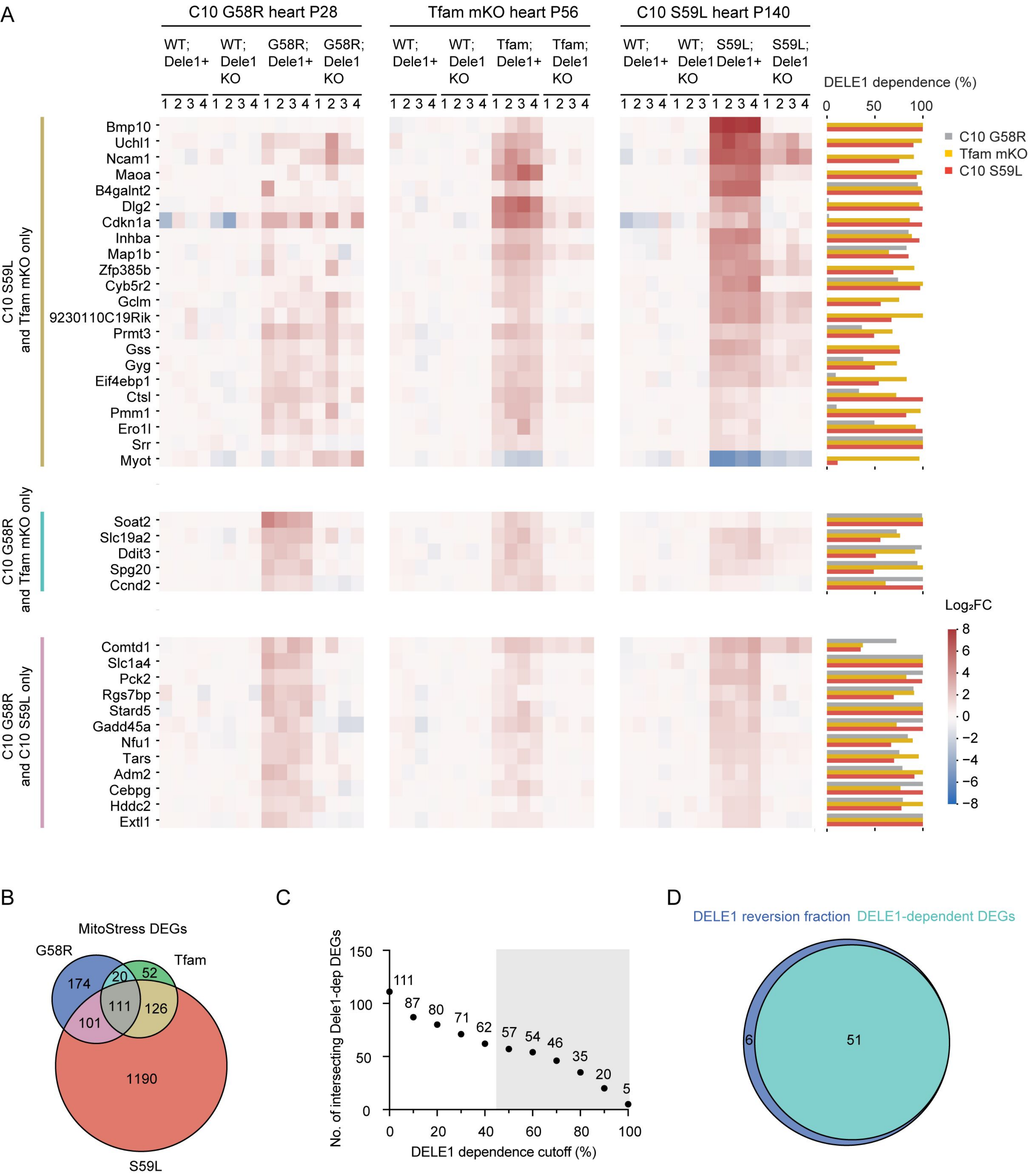

Supplemental Figure 9

105 and is also made available for use under a CC0 license.

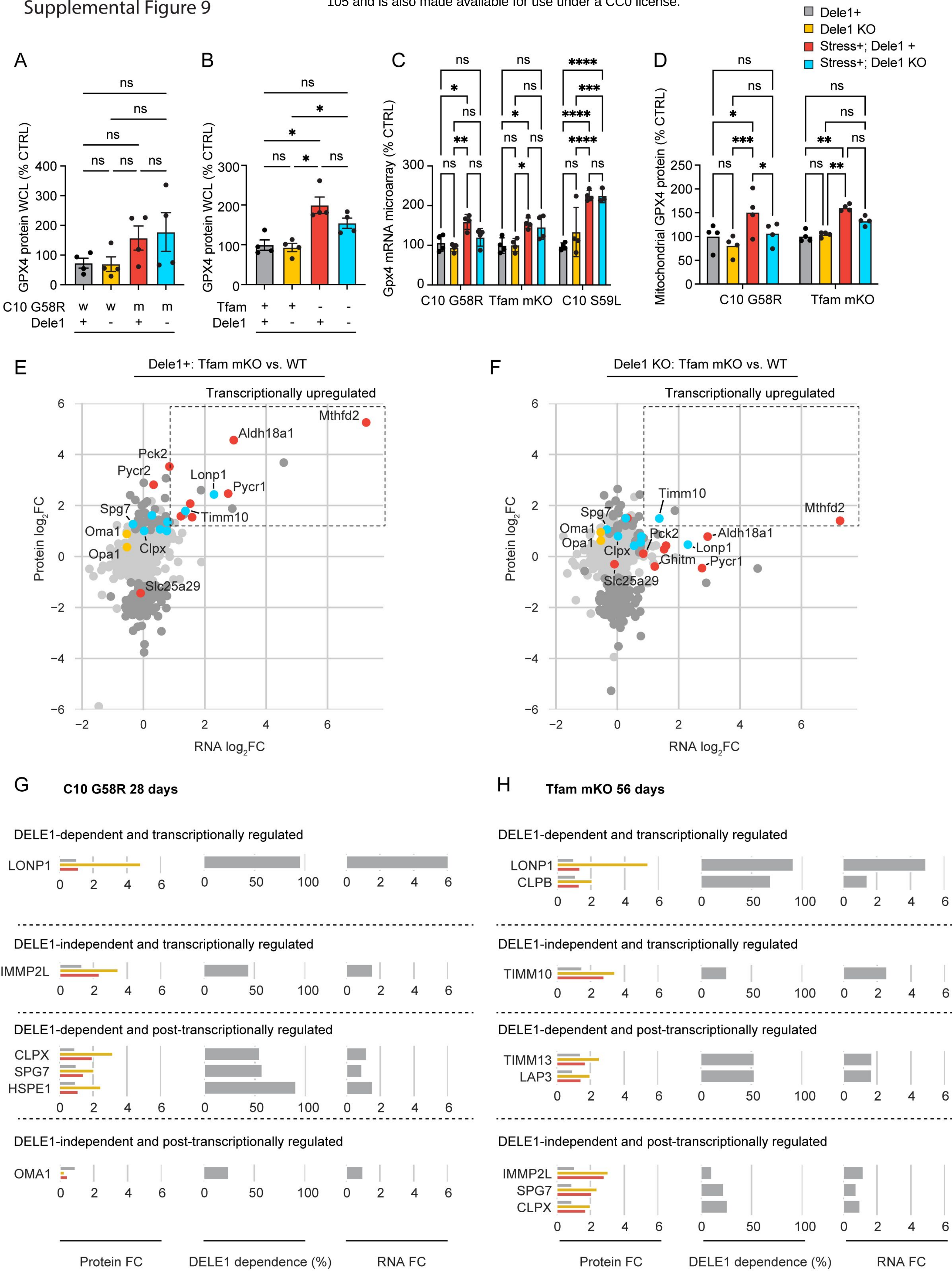

Supplemental Figure 10

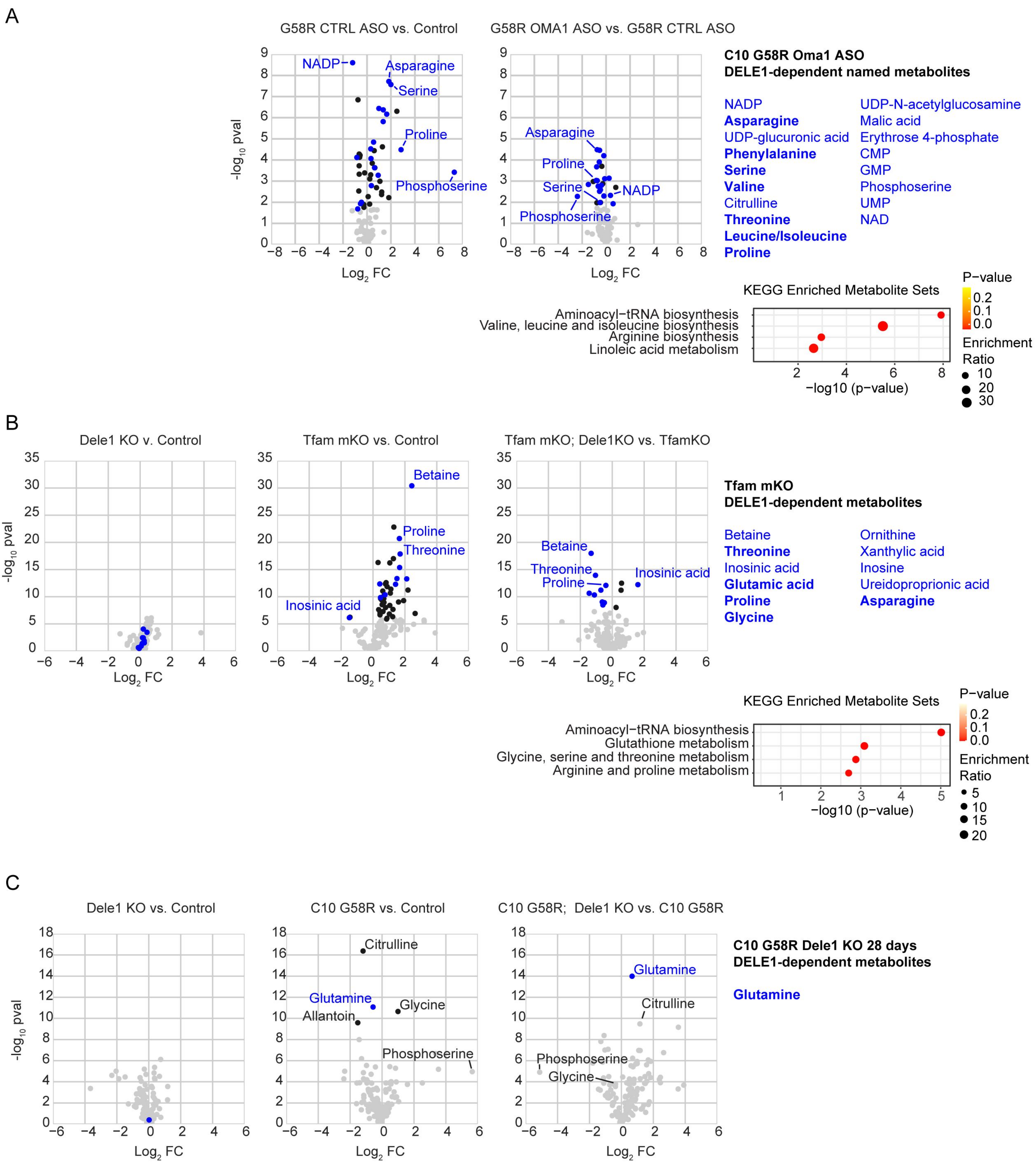

105 and is also made available for use under a CC0 license.

Supplemental Figure 11

A

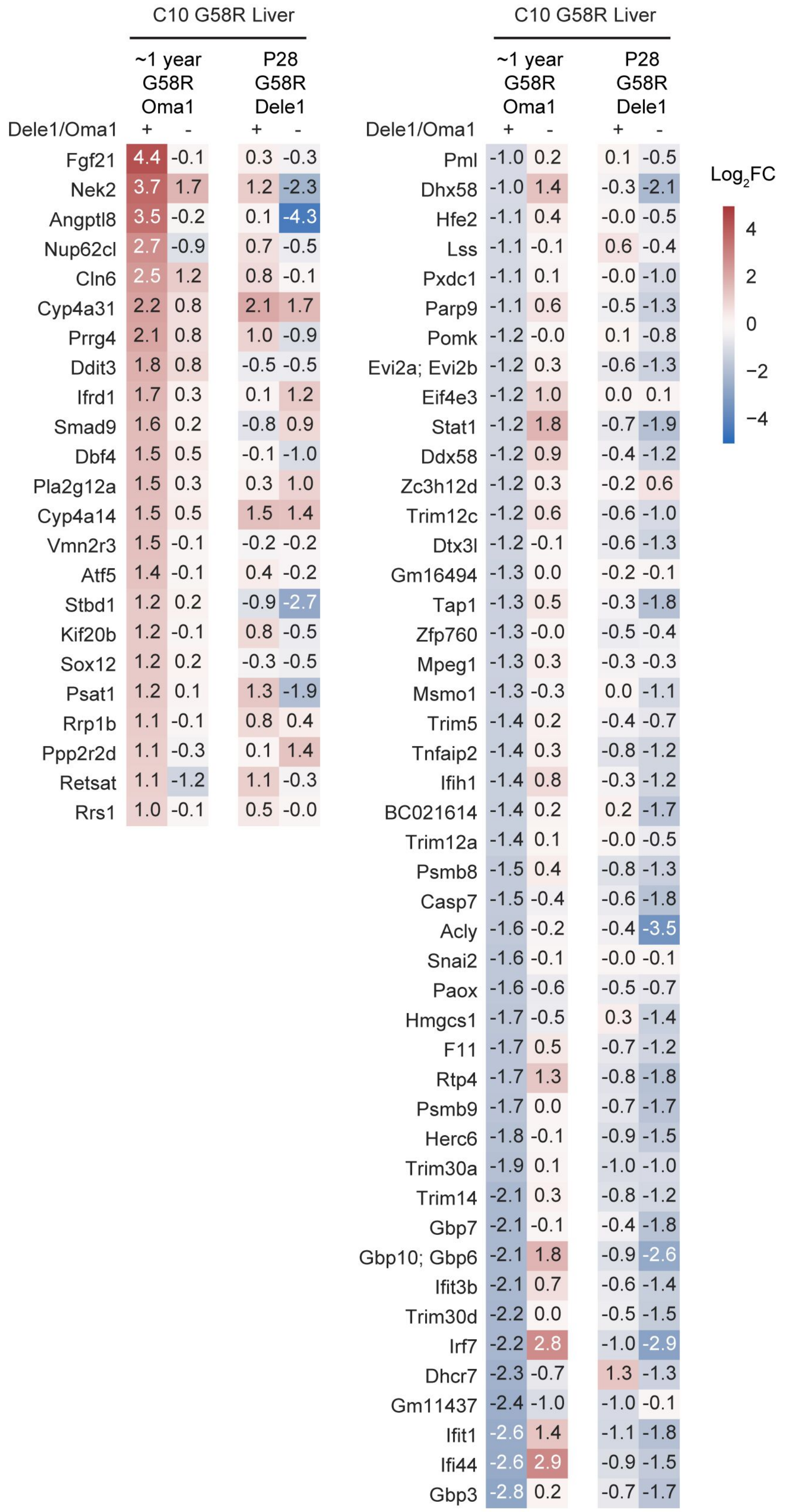

B

	Biological Process (Gene Ontology)			
GO-term	description	count in network	strength	false discovery rate
GO:0039528	Cytoplasmic pattern recognition receptor signaling pathwa in response to virus	3 of 10	2.0	0.0020
GO:0044790	Suppression of viral release by host	6 of 23	1.94	3.25e-07
GO:0031664	Regulation of lipopolysaccharide-mediated signaling pathway	5 of 28	1.77	3.57e-05
GO:0032727	Positive regulation of interferon-alpha production	4 of 26	1.71	0.00061

Supplemental Figure 12

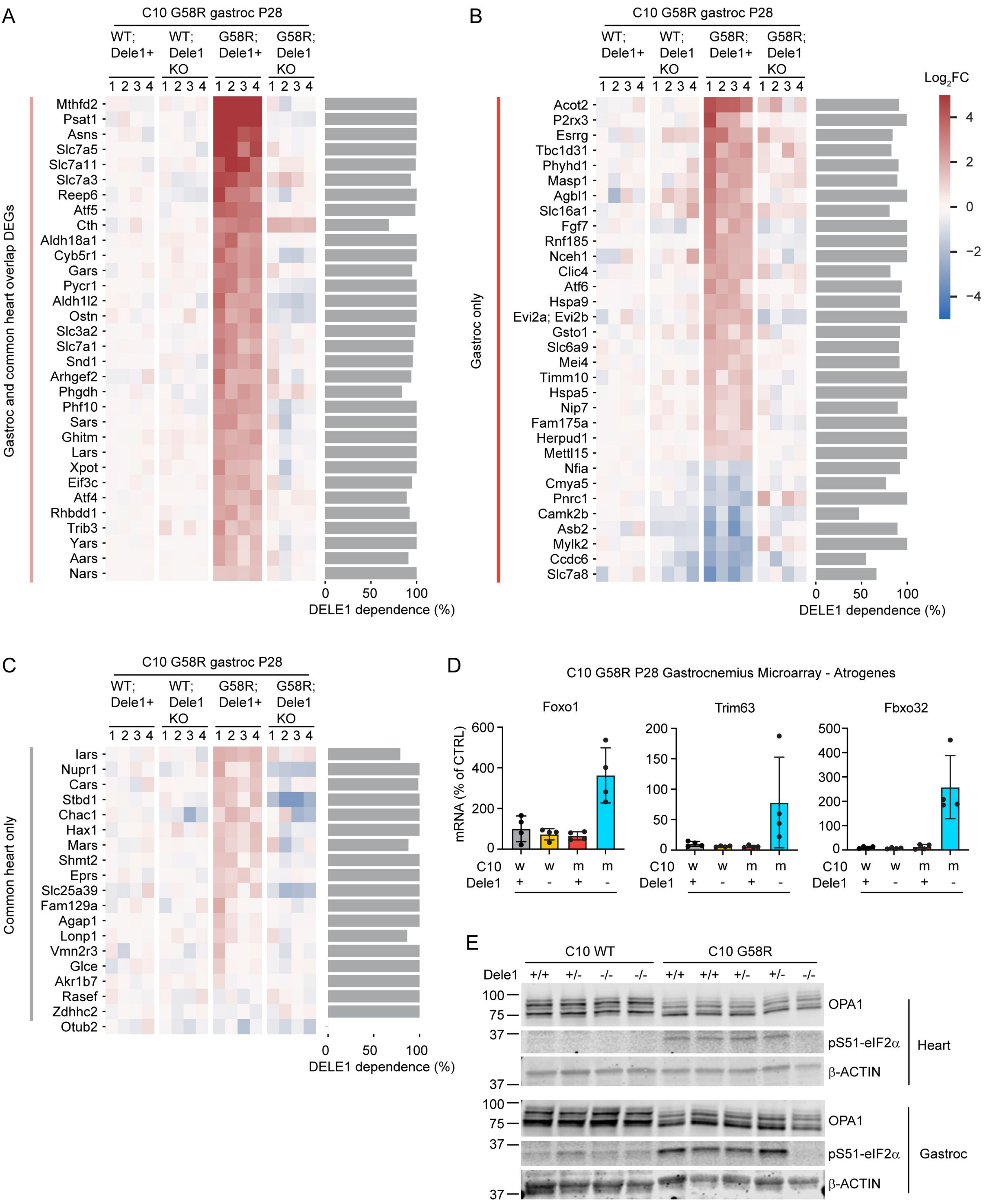

Supplemental Figure 13

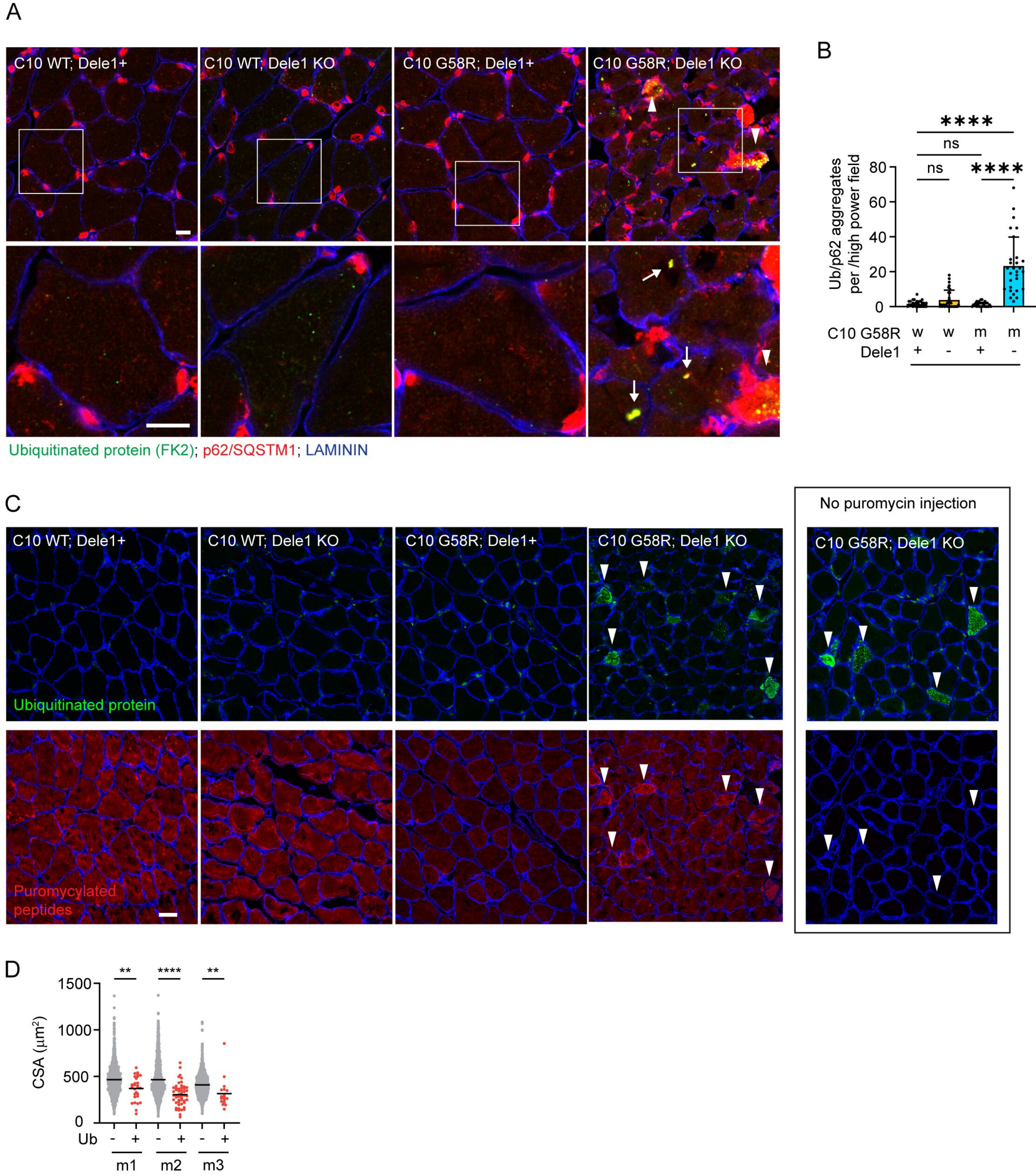

Supplement: 1 [file NIHPP2024.02.29.582673v1-supplement-1.pdf]
